# Cardiac and renal effects of liver cirrhosis in a growing animal model

**DOI:** 10.1590/ACB360806

**Published:** 2021-10-08

**Authors:** Ana Cristina Aoun Tannuri, Leiliane Somoggi Chavez, Juliana Xavier Guimarães, Josiane de Oliveira Gonçalves, Suellen Serafini, Gabriela Carvalho de Souza, Denise Maria Avancini Costa Malheiros, Vitor Ribeiro Paes, Uenis Tannuri

**Affiliations:** 1 Associate Professor. Pediatric Surgery Division - Pediatric Liver Transplantation Unit and Laboratory of Research in Pediatric Surgery (LIM 30) – Medical School - Universidade de São Paulo (USP) - Sao Paulo (SP), Brazil.; 2 Medical Researcher. Pediatric Surgery Division - Pediatric Liver Transplantation Unit and Laboratory of Research in Pediatric Surgery (LIM 30) – Medical School - Universidade de São Paulo (USP) - Sao Paulo (SP), Brazil.; 3 Medical Researcher. Pediatric Surgery Division - Pediatric Liver Transplantation Unit and Laboratory of Research in Pediatric Surgery (LIM 30) – Medical School - Universidade de São Paulo (USP) - Sao Paulo (SP), Brazil.; 4 Biologist. Laboratory of Research in Pediatric Surgery (LIM 30) - Universidade de São Paulo (USP) - Sao Paulo (SP), Brazil; 5 Biologist. Laboratory of Research in Pediatric Surgery (LIM 30) - Universidade de São Paulo (USP) - Sao Paulo (SP), Brazil.; 6 Medical Researcher. Pediatric Surgery Division - Pediatric Liver Transplantation Unit and Laboratory of Research in Pediatric Surgery (LIM 30) – Medical School - Universidade de São Paulo (USP) - Sao Paulo (SP), Brazil.; 7 Medical Researcher. Pediatric Surgery Division - Pediatric Liver Transplantation Unit and Laboratory of Research in Pediatric Surgery (LIM 30) – Medical School - Universidade de São Paulo (USP) - Sao Paulo (SP), Brazil.; 8 Associate Professor. Pathology Division - Medical School - Universidade de São Paulo (USP) - Sao Paulo (SP), Brazil.; 9 Head Professor. Pediatric Surgery Division - Pediatric Liver Transplantation Unit and Laboratory of Research in Pediatric Surgery (LIM 30) – Medical School - Universidade de São Paulo (USP) - Sao Paulo (SP), Brazil.

**Keywords:** Bile Ducts, Extrahepatic, Liver Cirrhosis, Models, Animal, Rats

## Abstract

**Purpose:**

To assess the biochemical, histological, histomorphometric and molecular effects of biliary duct ligation (BDL) induced liver cirrhosis in the heart and kidneys.

**Methods:**

Thirty-two weaning rats (21 days old, 50-70 g) underwent BDL and were divided in four groups (euthanasia after two, four, six, and eight weeks, respectively) and compared to control groups.

**Results:**

The animals’ hearts of group 3 were bigger than those of the control group (p=0.042), including thinner right ventricle wall, decreased internal diameter of ventricles, and increased perivascular collagen deposition in left ventricle, as well as increased interstitial collagen in right ventricle after six weeks. In the kidneys of groups 3 and 4, bilirubin impregnation in the tubules, hydropic degeneration, loss of nuclei and lack of plasmatic membrane limits were noted. Nitric oxide synthase (NOS) gene expressions were higher in group 1 (p=0.008), and endothelial nitric oxide synthase (eNOS) gene expressions were elevated in all experimental groups (p=0.008, p=0.001, p=0.022, and p=0.013, respectively). In the heart, a decreased expression of eNOS in group 1 (p=0.04) was observed.

**Conclusions:**

Liver cirrhosis leads to histological and histomorphometric alterations in the heart and kidneys, with changes in the NOS and eNOS gene expressions, that may suggest a role in the associated myocardial and renal manifestations.

## Introduction

Liver cirrhosis results from progressive replacement of normal parenchyma by fibrosis, nodule formation and the development of signs and symptoms of portal hypertension and organ failure. In children, the leading cause of cirrhosis requiring liver transplantation is biliary atresia^1^^,^ 2. Cirrhosis may also lead to manifestations in other organs, like heart^3^, lungs^4^, brain^5^, and kidneys^6^.

It was verified that patients with portal hypertension have increased both circulating and endothelial vasodilators^7^—such as nitric oxide (NO)^8^—, owing to a combination of impaired hepatic function and escape of vasodilators through portosystemic shunts^9^. This process contributes to the development of splanchnic arterial vasodilatation, leading to the development of a hyperdynamic syndrome with reduced central blood volume followed by baroreceptor-induced activation of the renin-angiotensin-aldosterone system (RAAS) and the sympathetic nervous system (SNS)^10^. This activation phenomenon causes renal vasoconstriction, which is intrinsically related to the development of the hepato-renal syndrome^11^.

In normal conditions, renal function is highly dependent on blood perfusion and tissue oxygenation that are mainly regulated by nitric oxide (NO) and nitric oxide synthase (NOS). NO is produced by NOS, present in three forms: neuronal (nNOS), endothelial (eNOS) and inducible by cytokines (iNOS)^12^. eNOS is normally expressed in tissues and produces NO in physiologic amounts. During ischemia-reperfusion injury, NO produced by eNOS improves the microcirculation by promoting vasodilatation and inhibition of platelet aggregation^13^.

Similarly, the apparent hypovolemia of cirrhotic patients leads to water and sodium retention by the kidney with subsequent increase in plasma volume, which, in addition to vasoconstriction mechanisms, promotes an increase in cardiac output and heart overload^14^. This is believed that it is the pathophysiological mechanism of cirrhotic cardiomyopathy, a condition that occurs in almost 50% of cirrhotic patients^15^, characterized by blunted contractile responsiveness to stress and/or altered diastolic relaxation with electrophysiological abnormalities, without a previous cardiac disease. Cirrhosis is associated with histological abnormalities in cardiomyocytes, namely edema, mild diffuse fibrosis, exudation, nuclear and cytoplasmic vacuolization, unusual pigmentation, and ventricular dilatation and hypertrophy^16^^-^ 18. Cirrhotic cardiomyopathy is etiologically independent from cirrhosis and it is implicated in the development of complications such as hepatic nephropathy^16^.

In fact, the renal and cardiac effects of cirrhosis are relatively well defined in adults, although it is not known if these findings are similar in cirrhotic children. Apart from the clinical studies, the use of experimental models to study liver cirrhosis and its effects is attractive. The common bile duct ligation (BDL) model in rats, both in adult and growing animals, presents several advantages, such as low cost, easy manipulation, and animal care, as well as simplicity of the surgical procedure. Our group has standardized this model in newborn and weaning rats, which has been shown to be a reliable model of biliary cirrhosis in developing organisms^17^^-^ 19.

Few studies have elucidated the renal and cardiac manifestations of liver cirrhosis in young organisms simulating children with biliary atresia. Therefore, the objective of the present study was to assess the renal and cardiac repercussions of BDL in a weaning rat model, by using biochemical, histological, histomorphometric and molecular analyses.

## Methods

The animals were cared according to the criteria outlined in the “Guide for Care and Use of Laboratory Animals”, prepared by the National Academy of Sciences. There were two study protocols that were reviewed and approved by the Animal Ethics Committee at our institution.

Fifty-two weaning Wistar rats of both sex (*Rattus norvegicus* Albinus, Rodentia, Mammalia), 21 days old, weighing 50-70 g, were utilized. All animals were housed under specific pathogen-free conditions, maintained in plastic cages (dimensions: 49 × 34 × 16 cm) with two littermates, on saw dust bedding, and subjected to a 12-hour light-dark cycle, in temperature 22°C ± 2°C, humidity-controlled environment (55%) and free access to purified water and food (Nuvilab CR-1 commercial food, Quimtia, Colombo, PR, Brazil). Cages were changed twice a week. The animals were acclimatized to the testing room at least three days before the experiments. Clinical signs such as food consumption, erection of the back hairs, harderian gland secretion (which occurs when the animals are irritated or alarmed), diarrhea and lethargy, were monitored and recorded one time per week during all the experimental periods. The rats that presented clinical signs of severe pain before the end of the experimental protocol were immediately euthanized by isoflurane overdose (Isoforine®, Cristália, Itapira, SP, Brazil).

The animals were divided into two groups: experimental (n=32) and control (n=20). In the experimental group, the animals underwent common bile duct ligation (BDL) and were divided into four subgroups with eight rats each, according to the time elapsed from BDL to euthanasia:

- Group 1: euthanasia two weeks after BDL;
- Group 2: euthanasia four weeks after BDL;
- Group 3: euthanasia six weeks after BDL;
- Group 4: euthanasia eight weeks after BDL.

Control animals were divided into four subgroups with five rats each, with ages matching the animals in the experimental group.

### Anesthesia and surgery

All researchers were appropriately qualified and competent through training in the utilized surgical procedures. The rats were anesthetized with an intraperitoneal injection of ketamine hydrochloride (Ketalar®) at 30 mg/kg and dexmedetomidine (Precedex®) at 10 mg/kg, with additional inhalation of isoflurane during the surgical procedure. Adequate depth of anesthesia was periodically verified by the absence of a nociceptive response to tail tip and interdigital pinch. Such a nociceptive response could either be any reactive movement or reflex (usually a pedal withdrawal reflex) or a noticeable rise in heart or breathing rate. In the case of inadequate analgesia, an additional intraperitoneal administration of 15 mg/kg of ketamine was performed.

The procedure consisted of a 2-cm midline incision in the upper abdomen starting immediately below the xiphoid process. The intestines and liver were exteriorized to allow for visualization of the bile ducts. Using mononylon 6.0, a double ligation of the common bile duct was performed followed by sectioning between the two ligations. The abdomen was closed with a continuous single suture using Mononylon 4.0. The animals were then cleaned and placed in recovery under analgesia, with water and food supplied *ad libitum*.

Upon completion of the experimental protocol, rats were weighed and euthanized by isoflurane overdose (Isoforine®, Cristália, Itapira, SP, Brazil), following the guidelines of the Ethics Committee on Animal Use of our institution. A wide longitudinal laparotomy was performed to expose the abdominal aorta, and arterial blood was collected for biochemistry tests. After sternotomy, the heart was removed. Liver and kidney samples were collected for molecular, histologic, and histomorphometric analyses.

Heart volume: the animals’ hearts were removed (at the junction with the large vessels) from the chest and placed in normal saline to wash the blood from the cardiac cavities. The heart was then immersed in a container filled with formaldehyde, and the total volume of the organ was calculated based on the volume of liquid displaced from the container.

### Biochemical tests

Serum levels of urea and creatinine of all animals were measured.

### Histological and histomorphometric analyses

For histological analysis, the specimens were kept for 24 hours in 10% formaldehyde. After fixation, the material was submitted to dehydration followed by paraffin embedding, and 4-µm-thick histological sections were stained with hematoxylin-eosin (HE) for general morphology studies and picrosirius red for identification of collagen fibers.

The histological slides were examined under light microscopy and blindly reviewed by two pathologists.

The analysis of liver parenchyma was performed in sections stained with HE to verify the alterations promoted by the BDL. The histological slides were blindly analyzed by two pathologists under an optical microscope, considering the degree of ductular proliferation. For biliary ducts count, five randomly chosen fields per slide containing at least one portal space were analyzed. The analyzed portal spaces were delineated with the help of a computer mouse.

Regarding the kidney, the renal parenchyma was prepared in transversal sections stained with HE and assessed for the presence of bilirubin impregnation in renal tubules and cytological alterations (hydropic degeneration, loss of nuclei and of plasmatic membrane limits). Five randomly chosen fields per slide were analyzed. Based on these findings, each field was classified according to the following criteria:

1. Absent;
2. Mild bilirubin impregnation;
3. Moderate bilirubin impregnation:
  - without cytological alterations;
  - with cytological alterations;
4. Intense bilirubin impregnation:
  - without cytological alterations;
  - with cytological alterations.

Concerning the heart, after fixation for a short time in 10% neutral buffered formalin, the heart was sectioned in the middle, immediately below the atrial-ventricular valves. The two halves were then placed again in 10% neutral buffered formalin for immersion fixation. Subsequently, they were embedded in paraffin blocks from which 4-µm-thick paraffin-tissue slides were prepared. These slides, a series of cross-sections through the ventricles, were stained with HE and picrosirius red.

The morphometric analysis was performed on five slides from each heart. Quantitative data were obtained with a computerized system for image analysis (NIS-Elements Advanced Research).

The following parameters were measured: left and right ventricles free wall thickness (200x magnification); and right and left ventricles internal diameters (400x magnification).

Collagen deposition in myocardial tissue was analyzed. The collagen accumulation in myocardial tissue was evaluated in slides stained with Sirius red. This analysis was performed by two different methods. The perivascular collagen was quantified by estimating the percentage of stained tissue (collagen fibers) within a total delimited area, with values expressed as percentage of collagen/µm^2^. Three random fields per ventricle containing at least one vessel each were captured. The vessel areas to be measured were delineated with the help of a computer mouse. A graphic resource was used to label the structures to be quantified (collagen fibers). Collagen and vessel space areas were measured in square micrometers. The collagen area was divided by the area of the region outlined in the vessel space, and the value obtained was expressed as the percentage of collagen (i.e., fraction of area).

The interstitial collagen was analyzed using a semi-quantitative scale. Four random fields on each slide were captured under 200x magnification. The presence of stained tissue (interstitial collagen fibers) per field was rated according to the following scale:

- 0: absent;
- 1: mild;
- 2: moderate;
- 3: severe.

### Molecular analysis

The expressions of NOS and eNOS in cardiac and renal tissues were analyzed by quantitative reverse transcription polymerase chain reaction (RT-PCR) method, according to previous publication from our laboratory^21^^,^ 22.

### Statistical analyses

For morphometric and heart volume data, a relation between these data and the weight of the animal was calculated and used for statistical comparisons. Statistical analyses were performed using the Statistical Package for the Social Sciences (SPSS) software 18.0 for Windows (SPSS, United States). The Shapiro-Wilk test was used to determine whether groups of data had Gaussian distribution. Continuous quantitative data were analyzed by t-test, one-way analysis of variance (ANOVA) and the Tukey post-hoc test to the two and three or more groups, respectively. Nonparametric data were analyzed by the Mann-Whitney test to compare two groups, and by Kruskal-Wallis and the Dunn post-hoc test to compare three or more groups, respectively. A two-tailed value of = 0.05 was considered statistically significant.

## Results

Six animals from the experimental groups (18.7%) died during the experiment. At the end of the experiment, each subgroup had a minimum of five surviving animals. All BDL animals showed dilation of the common biliary duct as a consequence of the distal obstruction that accentuated throughout time, with evident liver parenchyma alterations, ascites accumulation and splenomegaly.

**Figure 1:**
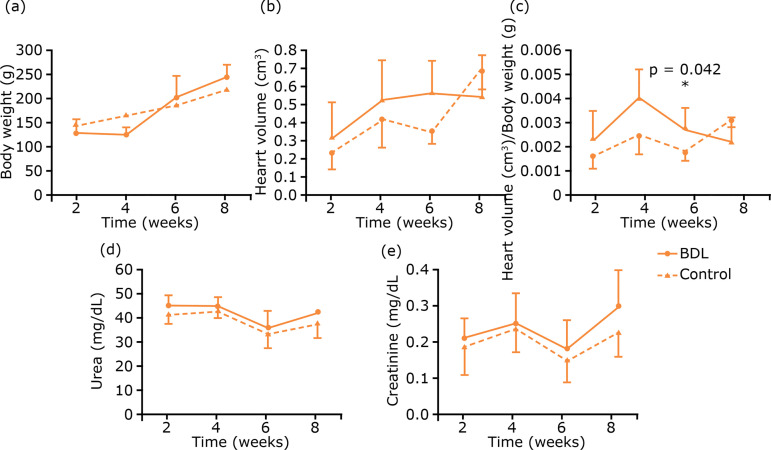
Results of morphometric and biochemical evaluations.

The results of biochemical tests, as well as the morphometric evaluations of body weight, heart volume and heart volume to body weight ratio, are shown in Fig. 1. There were no differences between serum levels of urea and creatinine in control and cirrhotic animals over the studied period. The hearts of cirrhotic animals were found to be larger than those of control ones six weeks after BDL (p= 0.042).

BDL: biliary duct ligation.

**Figure 2:**
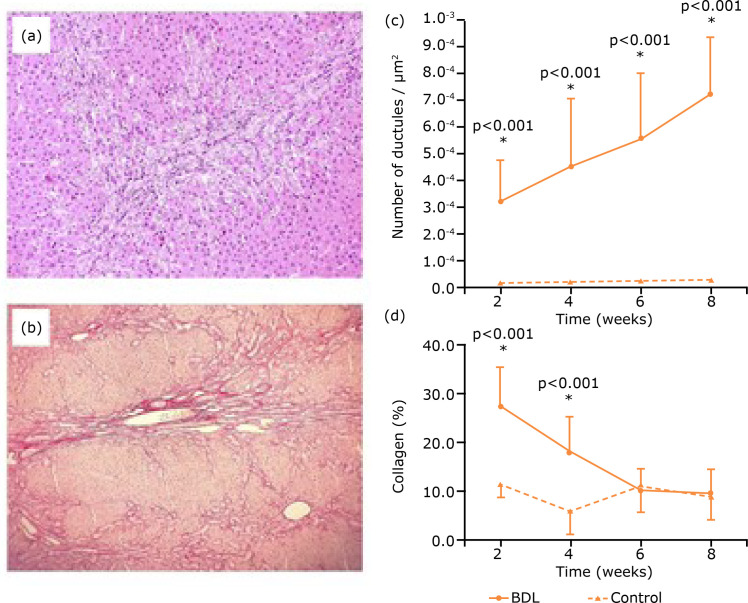
Histology and histomorphometry of the liver six weeks after BDL. **(a)** Portal tract with intense ductular proliferation (HE staining 200x magnification. **(b)** Representative section of Sirius red stained liver (200x magnification). **(c)** Results of the ductules counting of experimental and control groups. **(d)** Content of collagen on experimental and control animals.

BDL: biliary duct ligation; HE: hematoxylin-eosin.

Concerning the histology and histomorphometry of the liver, at light microscopy of HE stained sections, an intense ductular proliferation was noticed, and the intensity of this alteration was exacerbated proportionally to the time of observation after the BDL (Fig. 2).

**Figure 3:**
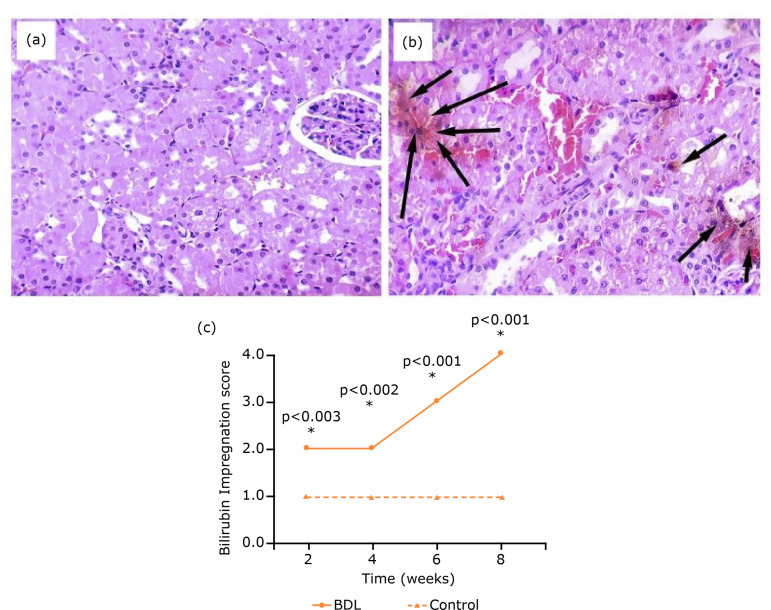
Histopathological changes and histomorphometry of the kidney after BDL. **(a)** Renal tubules and cytological alterations. **(b)** Presence of bilirubin impregnation in renal parenchyma. **(c)** Renal scores of experimental animals showing differences in comparison with control animals (HE staining, 400x magnification).

BDL: biliary duct ligation; HE: hematoxylin-eosin.

In the kidneys, histological findings were quite homogeneous among animals of the same group. In groups 1 and 2, small amounts of bilirubin were found in renal tubules. Animals in group 3 had marked bilirubin impregnation in renal tubules, as well as hydropic degeneration, loss of nuclei and absence of plasmatic membrane limits. Kidneys of animals in groups 3 and 4 showed intense impregnation with bilirubin, with formation of intracellular plugs, hydropic degeneration, loss of nuclei and absence of plasmatic membrane limits (Fig. 3). The histopathologic scores showed significant changes in comparison to control animals.

**Figure 4:**
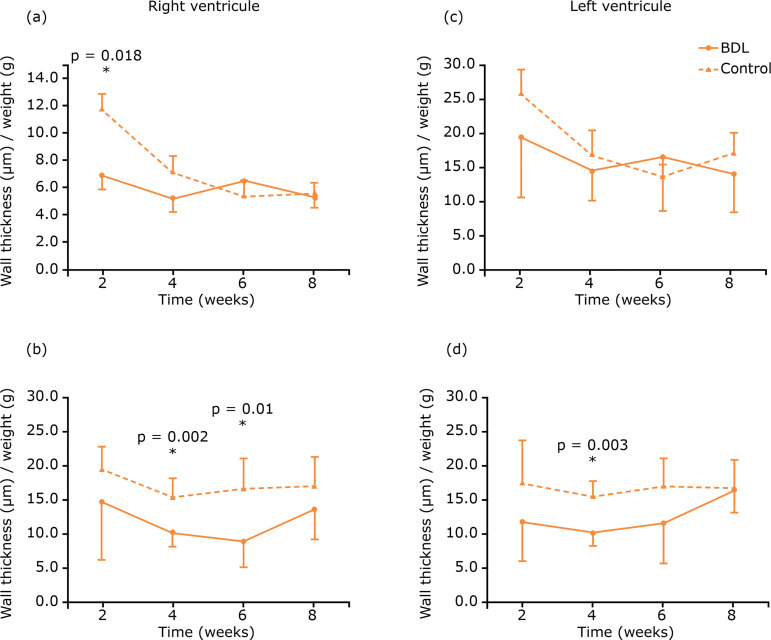
Histomorphometry of the heart. Differences between experimental and control groups were verified.

In the heart, reduced right ventricle wall was verified compared with that of controls (p=0.018), as expressed by the wall thickness to body weight ratio. The ratio between the internal diameter of both ventricles and the body weight was measured for all groups, and it was found that the right ventricle internal diameter in groups 2 and 3 was smaller than in controls (p=0.002 and p=0.01, respectively). For the left ventricle, this difference was observed only in group 2 (p=0.003) (Fig. 4).

**Figure 5:**
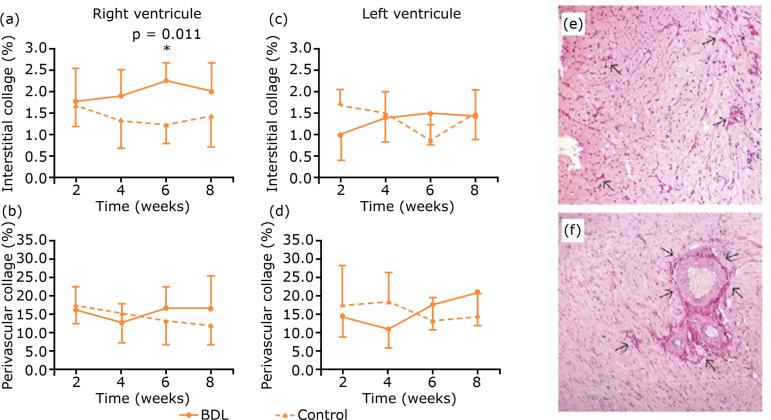
Results of the heart collagen studies. (**a, b, c and d)**. In the left ventricle of the experimental groups, perivascular collagen deposition increased over time although no difference was verified in comparison to controls. In the right ventricle, group 3 showed an increase *vs*. control animals (p=0.011). **(e)** Histopathological aspect of left ventricle – the arrows indicate collagen fibers. **(f)** The arrows indicate perivascular collagen fibers (HE staining, 400x magnification).

BDL: biliary duct ligation; HE: hematoxylin-eosin.

The study of heart collagen deposition showed no difference between groups for the right ventricle. However, in the left ventricle of the experimental groups, perivascular collagen deposition increased over time, being more marked in group 4 *vs*. group 1 (p=0.011), in group 3 *vs*. group 2 (p=0.008) and in group 4 *vs*. group 2 (p=0.001). In addition, there was no difference between groups concerning the amount of perivascular collagen in the right ventricle. Regarding interstitial collagen, group 3 showed an increase in the right ventricle vs. control animals (p=0.011) (Fig. 5).

**Figure 6:**
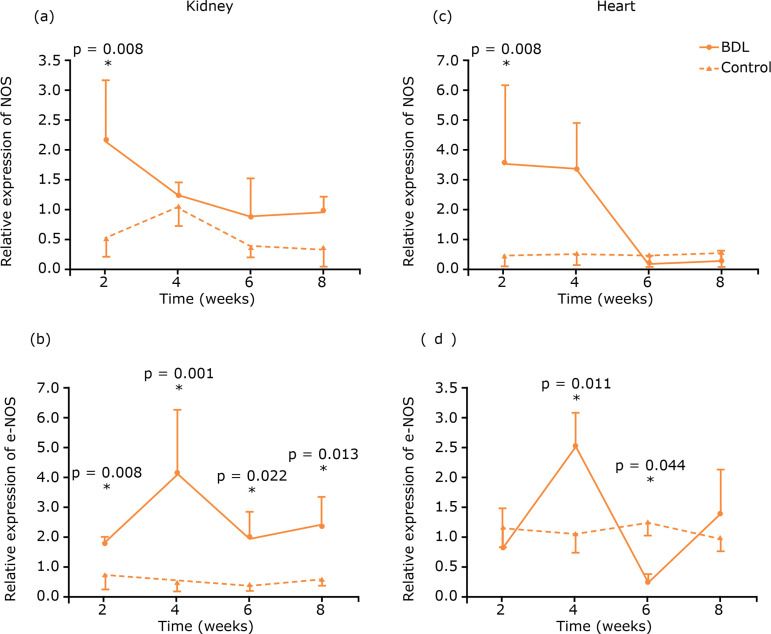
Analysis of relative expression of NOS and eNOS genes in the kidney and heart tissue.

BDL: biliary duct ligation; NOS: nitric oxide synthase; eNOS: endothelial nitric oxide synthase.

In the kidney, the NOS gene expression was higher in cholestatic animals than in controls two weeks after BDL (p=0.008). While the eNOS gene expression was similar in all experimental groups, it was always higher in these animals than in their corresponding controls (p=0.008, p=0.001, p=0.022 and p=0.013, respectively, for the comparison between groups 1, 2, 3 and 4 with controls). In the heart, no difference in total NOS was found. eNOS expression was lower in experimental *vs*. control animals two weeks after the procedure (p=0.04), but increased over time (group 1 *vs*. group 4: p=0.023), and it was similar to controls at eight weeks (Fig. 6).

## Discussion

While myocardial and renal repercussions of cirrhosis are clinically and experimentally well defined in adults, in children they are yet to be fully elucidated. Many studies have shown that children with biliary atresia in advanced cirrhotic stages develop renal dysfunction^17^,^18^ and, more recently, myocardial dysfunction^19^^-^ 21 mainly under stress.

The purpose of this study was to characterize renal and cardiac changes induced by biliary cirrhosis in young organisms, using the technique of common bile duct ligation in weaning rats. Previous studies have demonstrated that biliary ligation in adult mice and rats is a good model for studying cardiac^19^^-^ 21 and renal^19^^-^ 21 repercussions of cirrhosis.

The group has conducted previous studies using BDL in weaning rats and found a marked and fast development of histological and clinical signs of cirrhosis in these animals^19^,^20^. Despite the technical difficulties to deal with weaning rats, such as the small size of the structures, the friability of tissues (mainly liver lobes), through intensive training, low mortality rates related to the surgical procedure were reached. However, as time after BDL was prolonged (especially after four weeks), cirrhosis progression accounted for the increasing lethality.

Similarly, myocardial and renal dysfunctions in rats were expected to become more evident the longer the time after BDL.

The heart volume analysis allowed for an individualized measurement of the myocardial tissue, excluding the inside space of the chambers. The hearts of cirrhotic animals were bigger than those of controls, but showed a decreasing trend after eight weeks. This suggests that the cirrhotic heart initially develops hypertrophy, but after a certain point the muscular walls start to taper, and the contractile strength is consequently diminished.

The histomorphometric evaluation supported these findings. The ratio between the right and the left ventricle inner diameter, and total body weight remained stable in control animals as they grew up. In the BDL animals, there was an initial decrease in the inner diameter of both ventricles, suggesting myocardial hypertrophy. However, about the later groups of animals, the ventricle chambers showed a tendency to dilate, which suggests heart failure. In the control groups, the ventricle wall thickness seemed to decrease as the animal grew, stabilizing in the end. The experimental groups, on the other hand, did not exhibit this physiological tapering. It is a clear indication of the negative effects of cirrhosis on the growing heart.

In this study, it was also assessed if these cardiac histomorphometric changes were followed by myocardial fibrosis. In fact, progressive perivascular collagen deposition in the left ventricle was found, and the amount of interstitial collagen was consistently higher in all experimental animals *vs*. in their corresponding control groups.

Recently, the effects of the interactions of the liver-heart inflammatory axis and the cannabinoid 2 receptor (CB2-R) were studied in an experimental model of hepatic cardiomyopathy. It was shown that the CB2-R activation decreased serum TNF-alpha levels and improved cardiac dysfunction, myocardial inflammation, and oxidative stress, underlining the importance of inflammatory mediators in the pathology of hepatic cardiomyopathy^21^. It is possible to conclude that in young organisms a similar inflammatory process occurs, resulting later in deposition of perivascular and interstitial collagen, which probably has a role in cirrhotic myocardial dysfunction.

In other recent studies, it was shown that cardiac chronotropic dysfunction is mainly caused by increased cardiac NO synthesis^22^. NO modifies cardiac function through guanylyl cyclase dependent and independent mechanisms. Similarly, although total NOS expression was not altered by biliary ligation, a decrease in eNOS expression in the heart was initially observed in comparison to control animals. As cirrhosis progressed, the expression of this gene increased, a phenomenon that has been investigated in many studies^22^^-^ 24. It was demonstrated that NO resulting from eNOS activity has beneficial effects^22^^-^ 24. Therefore, it is possible that some protective or feedback mechanisms attempting to reduce the deleterious effects of cirrhosis in the heart lead to an increase in the eNOS gene expression, in late phases of liver disease.

In parallel, in the current study it was observed that cirrhosis leads to renal repercussions at the histological and molecular levels. The renal effects of BDL in adult rats were previously shown, with damage to renal function, reflected by increasing levels of urea and creatinine noticed two weeks after cholestasis induction, although no histological changes in renal parenchyma were noted^22^. Interestingly, in the present study the weaning rats showed a different renal response, with normal serial levels of urea and creatinine despite the histological changes in the kidney, which showed intense cellular impregnation of bilirubin, formation of intracellular plugs, hydropic degeneration, loss of nuclei and absence of plasmatic membrane limits.

Therefore, it seems that renal function is preserved in cirrhotic children in comparison to cirrhotic adults. In accordance with these findings, clinical practice shows that renal dysfunction is more frequent and intense in cirrhotic adults than in children^23^. This is the reason why the model of end-stage liver disease (MELD) score, used to rank adults eligible to liver transplant, considers creatinine serial levels, while the pediatric end-stage liver disease (PELD) score does not take creatinine levels into account^24^.

Some studies using a BDL adult rat model correlate oxidative stress and generation of free radicals with cirrhosis induced renal damage^25^,^26^. Similarly, changes in the NOS gene expression in the kidneys were observed in our model. Total NOS expression in BDL animals was higher than in controls after two weeks and tended to be higher than in controls in the other time points. Regarding eNOS expression, it was higher in all experimental animals in comparison to controls. Considering that iNOS renal activation correlates with renal dysfunction in adult rats undergoing BDL^27^, it is possible to conclude that this increased eNOS activation in young animals is related to the relatively better renal tolerance and to the milder functional repercussions as compared to adults. As mentioned before, eNOS activation is related to beneficial effects in other circumstances.

## Conclusions

It was shown that BDL-induced liver cirrhosis leads to progressive histological and histomorphometric alterations in the heart and kidneys of young animals, and this can be a good model for better elucidating cirrhotic nephropathy and cardiomyopathy in children. Additionally, changes in the NOS and eNOS genes expression in the heart and kidney suggest that NO plays an important role in the genesis of myocardial and renal repercussions of cirrhosis.
